# Disruption of STAT3-DNMT1 interaction by SH-I-14 induces re-expression of tumor suppressor genes and inhibits growth of triple-negative breast tumor

**DOI:** 10.18632/oncotarget.4054

**Published:** 2015-05-09

**Authors:** Hyo Jin Kang, Yong Weon Yi, Shu-Jie Hou, Hee Jeong Kim, Yali Kong, Insoo Bae, Milton L. Brown

**Affiliations:** ^1^ Department of Oncology, Georgetown University Medical Center, Washington, DC, USA; ^2^ Department of Radiation Medicine, Georgetown University Medical Center, Washington, DC, USA; ^3^ Center for Drug Discovery, Georgetown University Medical Center, Washington, DC, USA; ^4^ Lombardi Comprehensive Cancer Center, Georgetown University Medical Center, Washington, DC, USA

**Keywords:** SH-I-14, STAT3, DNMT1, acetylation, interaction

## Abstract

Epigenetic regulation of gene expression is an emerging target to treat several human diseases including cancers. In cancers, expressions of many tumor suppressor genes are suppressed by hyper-methylation in their regulatory regions. Herein, we describe a novel carbazole SH-I-14 that decreased the level of the acetyl-STAT3 at the K685 residue. Mutation analysis revealed that SH-I-14 disrupted STAT3-DNMT1 interaction by removing acetyl group from K685 of STAT3. Finally, the inhibition of STAT3-DNMT1 interaction by SH-I-14 resulted in re-expression of tumor suppressor genes such as VHL and PDLIM4 through de-methylation of their promoter regions. In addition, SH-I-14 showed anti-proliferative effect in triple-negative breast cancer (TNBC) cell lines *in vitro* and anti-tumor effect in a mouse xenograft model of MDA-MB-231 tumor. Taken together, our results suggest that targeting acetyl-STAT3 (K685) provides potential therapeutic opportunity to treat a subset of human cancers.

## INTRODUCTION

Signal transducer and activator of transcription 3 (STAT3), a member of STAT family, is a transcriptional regulator that mediates transduction of extracellular signals to the nucleus in response to various cytokines [1]. STAT3 is activated by phosphorylation of Y705 residue that triggered by cytokine receptor associated kinases such as Janus kinases (JAKs) or receptor tyrosine kinases upon ligand binding [1]. STAT3 is widely recognized as a potential drug target because: a) activation and/or overexpression of STAT3 is widely associated with many human cancers [2–4]; b) it induces tumor-promoting inflammation and suppresses anti-tumor immunity [2]; and c) it induces various anti-apoptotic and/or pro-proliferative gene expressions [2].

The regulation of STAT3 activity through reduction of phosphorylation [2, 4] and various inhibitors that reduce the phosphorylation of STAT3 are under active investigation [3, 4]. By reducing the phospho-STAT3, these inhibitors suppress the STAT3-mediated transcriptional activation of anti-apoptotic and/or pro-survival proteins [3, 4]. Recent advances in STAT3 biology, however, suggest new aspects of STAT3 function in cancers. First, STAT3 has been reported to have the repressive effects on the expression of tumor suppressor (TS) or pro-apoptotic genes [5–11]. Second, it has been reported that STAT3 is regulated by multiple acetylation [12] and acetylation of STAT3 at K685 and is an important player in DNA methylation of promoter regions for TS genes through interaction with DNA (cytosine-5)-methyltransferase 1 (DNMT1) [13, 14]. It was also found that acetylation of STAT3 is elevated in tumors and contributes to tumor progression by inducing DNA methylation [13]. Therefore, acetyl-STAT3 could be a potent target for tumor treatment and several small-molecules that inhibit the acetylation of -STAT3 have been reported [13, 15, 16].

Since *9H*-carbazole was first described in 1872 [17], numerous natural carbazoles have been identified from various plants and microorganisms [17, 18]. After discovering antitumor activity of ellipticine in the 1960s, antitumor activity of carbazoles has been widely studied [18]. However, few of them have advanced to clinical investigation [18]. As an attempt to discover novel potent small molecules with anti-tumor activity, recently we synthesized a series of carbazoles with fluorescent moiety [19, 20]. One of them, SH-I-14 has been identified as an inhibitor of phospho-STAT3 (Y705) by induction of protein-tyrosine phosphatase non-receptor type 6 (PTPN6/SHP-1) expression [20]. Here, we further evaluated SH-I-14 as an inhibitor of acetyl-STAT3 (K685). Inhibition of acetyl-STAT3 by SH-I-14 resulted in disruption of STAT3-DNMT1 interaction, de-methylation of TS genes’ promoter regions in triple-negative breast cancer (TNBC) cells *in vitro*, re-expression of TS genes and inhibition of TNBC tumor growth *in vivo*. Our findings further suggest that acetylation of STAT3 (K685) is a novel therapeutic target to treat human cancers.

## RESULTS

### SH-I-14 inhibits the proliferation of TNBC cells

**Figure 1:**
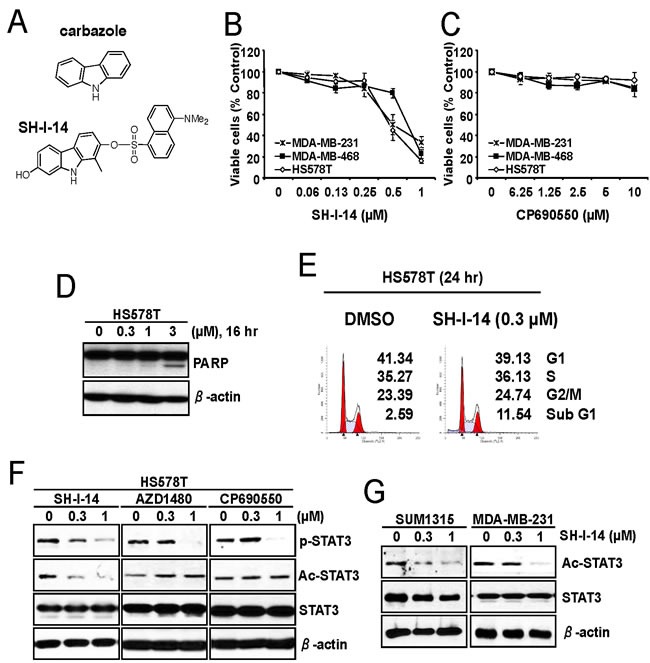
SH-I-14 represses the proliferation of TNBC cells and reduces acetylation of STAT3 **A.** Structures of carbazole and SH-I-14. **B.** and **C.** The anti-proliferative effect of SH-I-14 and CP690550 in TNBC cells. The cells were treated with increasing concentrations of SH-I-14 **B.** or CP690550 **C.** for ~72 hr and the viable cells were measured by MTT assay. Data from two independent experiments performed in triplicate are shown as mean ± SEM. **D.** SH-I-14 induced PARP cleavage in a dose-dependent manner. HS578T cells were treated with increasing concentration of SH-I-14 for 16 hr and the cells (both attached and floating) were harvested for western blot analysis as indicated. β-actin was used as a loading control. **E.** SH-I-14 increased sub-G1 population of cells. HS578T cells were treated with SH-I-14 for 24 hr and the cells (both attached and floating) were harvested for cell cycle analysis as described in Materials and Methods. **F.** Effects of SH-I-14 on the level of phospho- and acetyl-STAT3 in HS578T cells. The cells were treated with different concentrations of SH-I-14, AZD1480, or CP690550 for 24 hr and their lysates were subjected to western blot analysis with indicated antibodies. **G.** SH-I-14 inhibits acetyl-STAT3 in SUM1315MO2 and MDA-MB-231 cells. Cells were treated with increasing amount of SH-I-14 for 24 hr and western blot analysis was performed with indicated antibodies. β-actin was used as a loading control. Abbreviations: Ac, acetyl; and p, phospho; SUM1315, SUM1315MO2.

First, we determined the cytotoxic effect of SH-I-14 (Figure 1A) in three TNBC cell lines. The cells were treated with a range of concentrations of SH-I-14 for ~72 hr and the viable cells were measured by MTT assay. As reported recently [20], all these TNBC cells tested were sensitive and more than 60% reduction of viable cells was observed at 1 μM (Figure 1B). Indeed, SH-I-14 showed the antiproliferative effect on a broad range of TNBC cell lines tested [20]. On the contrary, a JAK1/2 inhibitor CP690550 [21] had no significant effect on the proliferation of TNBC cells up to 10 μM (Figure 1C).

The cytotoxic effect of SH-I-14 was further analyzed by western blot and cell cycle analysis in HS578T cells. As shown in Figure 1D, SH-I-14 induced cleavage of poly(ADP-ribose) polymerase (PARP), a hallmark of apoptotic cell death [22], in a dose-dependent manner within 16 hr post-treatment. In addition, 0.3 μM of SH-I-14 induced a modest increase of sub-G1 population in cell cycle analysis (Figure 1E).

### SH-I-14 reduces acetylation of STAT3 (K685)

In our previous study, we found that SH-I-14 inhibits phospho-STAT3 (Y705) by induction of PTPN6/SHP-1 expression [20]. Since it has been reported that PTPN6/SHP-1 is epigenetically repressed by STAT3-DNMT1 [7] and acetylation of STAT3 (K685) is critically to bind DNMT1 [13], we further determined the effect of SH-I-14 on the acetylation of STAT3 (K685) by western blot analysis performed in lysates from HS578T cells treated with a range of concentrations of SH-I-14, a JAK2 inhibitor AZD1480 [23], or a JAK1/2 inhibitor CP690550. As shown in Figure 1F, all these compounds reduced the phospho-STAT3 (Y705) in a dose-dependent manner. Interestingly SH-I-14 also decrease the acetyl-STAT3 (K685) level in HS578T cells in a dose-dependent manner (Figure 1F). Contrarily, the JAK2 (AZD1480) or JAK1/2 (CP690550) inhibitors had little or no effect on the acetyl-STAT3 (K685). Notably, the acetyl-STAT3 (K685) was detectable in all TNBC cell lines tested (Supplementary information, Figure S1) and SH-I-14 also decrease the level of acetyl-STAT3 in two additional TNBC cell lines, SUM1315MO2 and MDA-MB-231 in a dose-dependent manner (Figure 1G).

### SH-I-14 disrupts DNMT1-STAT3 interaction

**Figure 2:**
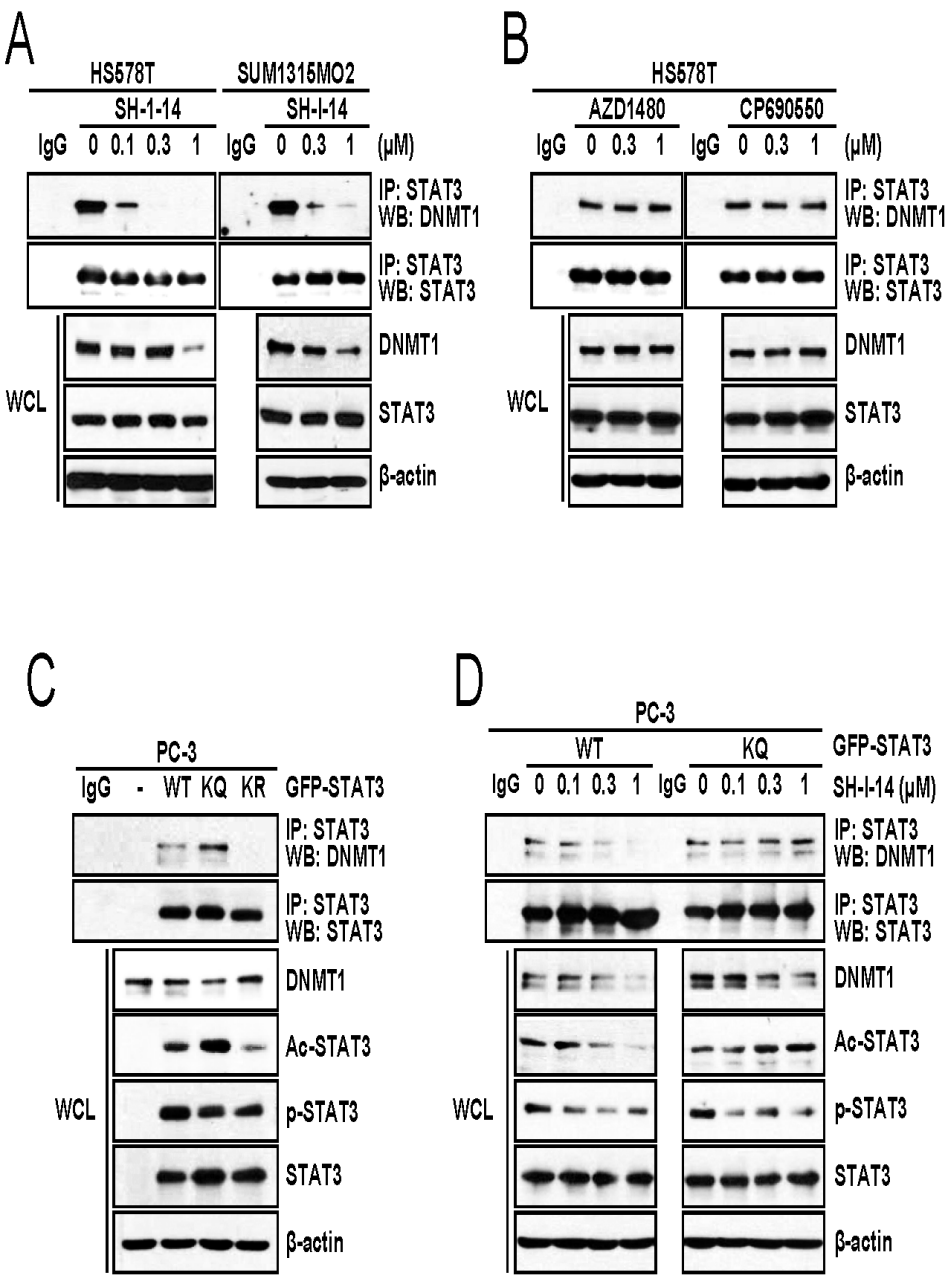
SH-I-14 disrupts the STAT3-DNMT1 interaction **A.** SH-I-14 inhibits the STAT3-DNMT1 interaction in HS578T and SUM1315MO2. Cells were treated with increasing amount of SH-I-14 for 24 hr and STAT3-bound complexes were precipitated by anti-STAT3 antibody. Western blot analysis was performed with indicated antibodies. **B.** JAK1/2 inhibitors do not disrupt STAT3-DNMT1 interaction. HS578T cells were treated with increasing amount of compounds for 24 hr and immunoprecipitation/western blot analysis was performed as in **A.**. **C.** STAT3-DNMT1 interaction is dependent on the acetylation of STAT3 at K685. GFP-tagged wild type STAT3, STAT3 (K685Q; KQ), or STAT3 (K685R; KR) was transfected into PC-3 cells. After immunoprecipitation by anti-STAT3 antibody, western blot analysis was performed with indicated antibodies. **D.** SH-I-14 disrupts wild type STAT3-DNMT1 interaction but not STAT3 KQ-DNMT1 interaction. PC-3 cells were transfected with either GFP-STAT3 WT or GFP-STAT3 KQ and STAT3 proteins were immunoprecipitated by anti-STAT3 antibody. β-actin was used as a loading control. Abbreviations: WT, wild type STAT3; KQ, STAT3 (K685Q); KR, STAT3 (K685R); WCL, whole cell lysates; IP, immunoprecipitation; and WB, western blot.

Given that acetylation of STAT3 at K685 is crucial for binding of STAT3 to DNMT1 [13], we determined the STAT3-DNMT1 interaction in the presence of SH-I-14. HS578T cells were treated with increasing concentrations of SH-I-14 for 24 hr and STAT3-bound proteins were immunoprecipitated by anti-STAT3 antibody. Western blot analysis demonstrated that STAT3 bound to DNMT1 in HS578T cells. Under this condition, STAT3-DNMT1 interaction was abolished by SH-I-14 as low as 0.3 μM (Figure 2A, left). Although the highest concentration (1 μM) of SH-I-14 reduced the level of DNMT1 protein, the disruption of STAT3-DNMT1 interaction by SH-I-14 occurred at much lower concentration (0.1 μM) that did not affect the level of DNMT1. SH-I-14 also disrupted STAT3-DNMT1 interaction in SUM1315MO2 cells (Figure 2A, right). On the contrary, the JAK2 inhibitor (AZD1480) or JAK1/2 inhibitor (CP690550) did not affect STAT3-DNMT1 interaction (Figure 2B).

We further analyzed the importance of acetyl-STAT3 (K685) in the SH-I-14-mediated disruption of STAT3-DNMT1 interaction. Expression vector for Green fluorescent protein (GFP)-tagged wild type STAT3, STAT3 (K685Q), or STAT3 (K685R) was transfected into a STAT3-null cell, PC-3 [24]. GFP-STAT3 proteins were immunoprecipitated by anti-STAT3 antibody and immune complexes were analyzed by western blot analysis. As expected, overexpressed GFP-STAT3 could interact with endogenous DNMT1 in PC-3 cells (Figure 2C). As reported [13], the acetylation-defective mutant (K685R; KR) [23] abrogated GFP-STAT3-DNMT1 interaction. On the contrary, mutation of K685Q (KQ), which mimics the acetylation [25], slightly increased the GFP-STAT3-DNMT1 interaction (Figure 2C). Consistent with previous report [13], under this experimental setting, GFP-STAT3 (KQ) was resistant to SH-I-14-mediated disruption of STAT3-DNMT1 interaction compared to wild type GFP-STAT3 (Figure 2D). However, SH-I-14 reduced phosphorylation of Y705 residue regardless of mutation at K685. These results suggest that disruption of STAT3-DNMT1 interaction by SH-I-14 is dependent on the de-acetylation of STAT3 at K685.

**Figure 3:**
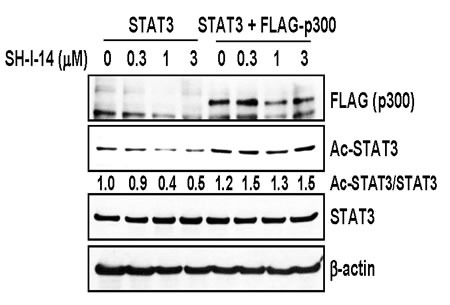
Overexpression of p300 reversed SH-I-14-mediated deacetylation of STAT3 HEK293T cells were transfected with STAT3 expression vector in the absence or presence of p300 expression vector. The cells were further treated with increasing amount of SH-I-14 for 24 hr and western blot analysis was performed with indicated antibodies. β-actin was used as a loading control.

Since the histone acetyltransferase p300 acetylates STAT3 (K685) [25, 26], we further tested the effect of p300 overexpression on the SH-I-14-mediated de-acetylation of STAT3. HEK293T cells were transfected with STAT3 expression vector in the presence or absence of FLAG-p300 expression vector, further treated with increasing amount of SH-I-14, and then analyzed by western blot. As reported [25, 26], overexpression of p300 increased the acetyl-STAT3 (K685) and overexpression of p300 suppressed de-acetylation of STAT3 by SH-I-14 (Figure 3).

### SH-I-14 induces the re-expression of TS genes via demethylation of DNA

**Figure 4:**
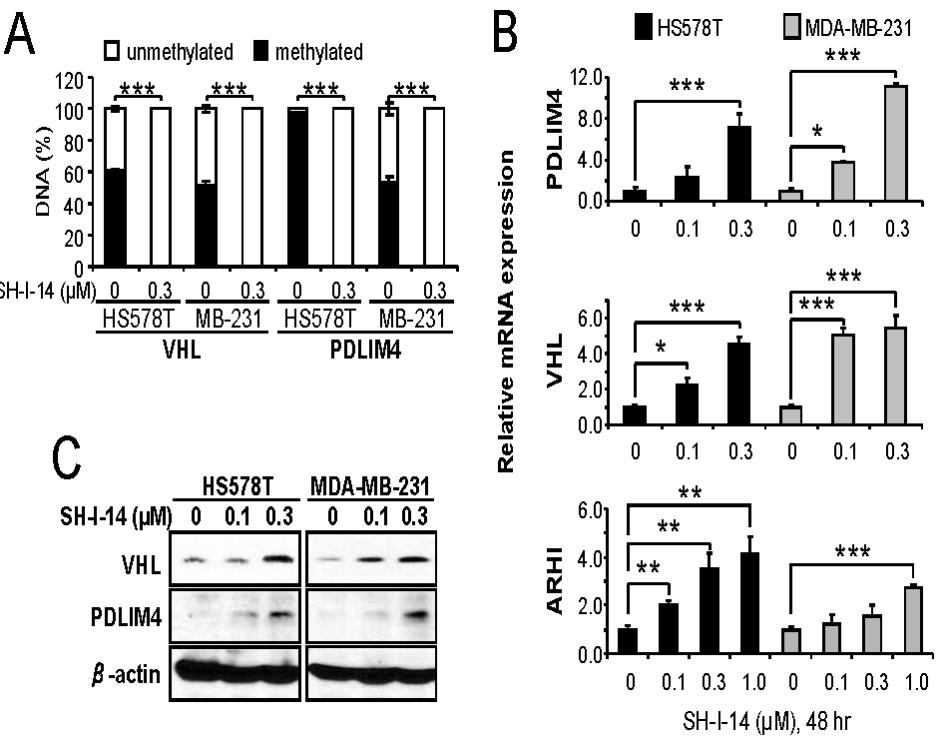
SH-I-14 induces demethylation and re-expression of tumor suppressor genes Cells were treated with SH-I-14 for 48 hr and subjected to DNA methylation assay **A.**, qRT-PCR for mRNA expression **B.**, or western blot analysis **C.**. **A.** Genomic DNAs from the cells treated with SH-I-14 were treated with methylation-specific restriction enzymes followed by qRT-PCR to determine the methylation status of each promoter. **B.** The cDNAs, synthesized from total RNAs of the cells incubated with SH-I-14, were used for qRT-PCR to measure the level of mRNAs. GAPDH level was used to normalize the level of cDNAs. (A~B) *P < 0.05; **P < 0.01; and ***P < 0.001. **C.** The lysates from the cells treated with SH-I-14 for 48 hr were analyzed by western blot with indicated antibodies. β-actin was used as a loading control.

The STAT3-DNMT1 interaction has been reported to be important for DNA methylation in the promoter region of TS genes [7, 13, 14]. Since SH-I-14 disrupts STAT3-DNMT1 interactions by decreasing the level of acetyl-STAT3 (K685), we further determine the effect of SH-I-14 on the methylation of DNA. The DNA methylation status of a panel of specific primers for 22 TS gene promoters was analyzed in HS578T cells treated with 0.3μM of SH-I-14 for 48 hr. As results, we identified four TS genes, that were de- methylated by SH-I-14 in HS578T cells, including retinoic acid receptor beta (RARB), neurogenin 1 (NEUROG1), PDZ and LIM domain 4 (PDLIM4), and Von Hippel-Lindau tumor suppressor (VHL) (data not shown). We further analyzed the methylation status of these promoters in two TNBC cells. HS578T and MDA-MB-231 cell lines were treated with 0.3μM of SH-I-14 for 48 hr and promoter methylations were analyzed by specific primers for each gene’s promoter. In both cells, the promoter regions of VHL and PDLIM4 genes were highly methylated and SH-I-14 near commonly de-methylated these promoters (Figure 4A).

To further verify the consequence of promoter de-methylation, mRNA and protein expression of VHL and PDLIM4 was analyzed by qRT-PCR and western blot analyses. First, the cells were treated with an increasing amount of SH-I-14 for 48 hr and RNAs from these cells were subjected to qRT-PCR analysis. De-methylation of VHL and PDLIM4 gene promoters by SH-I-14 resulted in reactivation of mRNA expression of these genes in both HS578T and MDA-MB-231 in a dose-dependent manner (Figure 4B). Another TS gene, a Ras homologue member I (ARHI), that regulated by STAT3-DNMT1-dependent methylation in ovarian cancer cells [14] was also induced by SH-I-14 (Figure 4B). Next, lysates from the cells treated with different concentration of SH-I-14 for 48 hr were used to determine the expression of these proteins. Consistent with mRNA expression, the levels of VHL and PDLIM4 proteins were increased by SH-I-14 in a dose-dependent manner (Figure 4C).

### SH-I-14 reduces tumor growth *in vivo*

**Figure 5:**
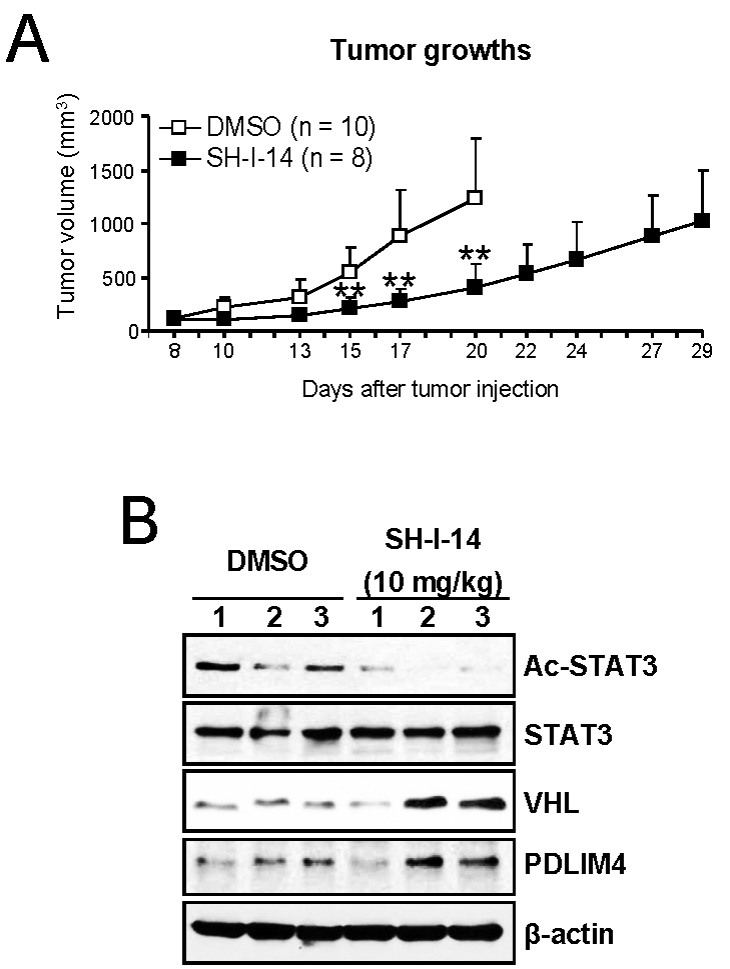
SH-I-14 reduces the *in vivo* growth of tumors in xenograft mice bearing tumors of MDA-MB-231 cells **A.** SH-I-14 (10 mg/kg) or DMSO was injected into peritoneal cavity of mouse with MDA-MB-231 tumors three times per week from 8-days after tumor cell injection. The control mice were euthanized earlier due to large tumor size. Tumor volumes were measured as described in Materials and Methods. Data are shown as mean±SD. **P < 0.01. **B.** SH-I-14 reduces acetyl-STAT3 and induces the expression of VHL and PDLIM4 protein *in vivo*. The MDA-MB-231 xenograft mice (3 mice per group) were administered with 10 mg/kg of SH-I-14 daily for 5 days and lysates from each tumor were subjected to western blot analysis as indicated. β-actin was used as a loading control.

To address whether SH-I-14 affects tumor growth *in vivo*, SH-I-14 (10 mg/kg) was administrated three times per week to athymic nude mice bearing MDA-MB-231 human TNBC tumors. As shown in Figure 5A, SH-I-14 markedly reduced the tumor growth. No apparent loss of body weights was observed during the treatment (Supplementary information, Figure S2).

To determine the level of markers in tumor samples, we administered SH-I-14 to MDA-MB-231 xenograft mice daily for 5-days and western blot analysis was performed with lysates from tumor samples. As shown in Figure 5B, the level of acetyl-STAT3 (K685) was reduced in tumor samples treated with SH-I-14 compared to tumor samples treated with DMSO control. In addition, the level of two TS proteins, VHL and PDLIM4, was increased in SH-I-14-treated tumor samples.

## DISCUSSION

Here we report a small molecule compound, SH-I-14, that inhibits *in vivo* tumor growth in a human TNBC xenograft model. SH-I-14 inhibits STAT3-DNMT1 interaction by reducing the acetylation of STAT3 (K685). In addition, SH-I-14 induces re-expression of TS genes, VHL and PDLIM4, through de-methylation of DNA in their promoter regions. Administration of SH-I-14 markedly reduced the *in vivo* tumor growth in a mouse xenograft model-bearing tumor of human MDA-MB-231 cells. It was also observed that de-acetylation of STAT3 and expression of VHL and PDLIM4 was induced by SH-I-14 in tumors from xenograft mice.

Acetylation of STAT3 was first reported in 2005 [24]. Subsequently, several studies have revealed that acetylation/de-acetylation of STAT3 at multiple residues is mediated by histone acetyltransferase (HATs) CBP and p300, histone deacetylases (HDACs) 1, 2, and 3, and sirtuin (SIRT) 1 in response to various extracellular signals [13, 25–30]. Among multiple acetylation sites of STAT3, acetylation of K685 is most well studied. Studies with acetylation-defective mutant STAT3 (K685R) demonstrated that this mutant inhibits dimerization, nuclear translocation and DNA binding of STAT3 and reduces expression of its target genes [24, 25]. More recently, acetyl-STAT3 (K685) has been reported to regulate DNA methylation through interaction with DNMT1 [13]. Consistent with these findings, SH-I-14 reduced acetyl-STAT3 (K685) and disrupted the STAT3-DNMT1 interaction. Disruption of STAT3-DNMT1 interaction by SH-I-14 is dependent on the acetylation of K685, since SH-I-14 could not disrupt the interaction between DNMT1 and the acetyl-mimic mutant STAT3 (K685Q).

**Figure 6:**
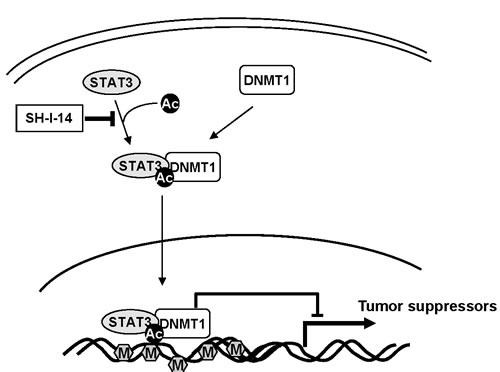
Schematic diagram of potential mechanism of SH-I-14 Abbreviations: Ac, acetylation; M, methylation.

STAT3-DNMT1 interaction implicates new functional roles of STAT3 in epigenetic gene silencing in human cancer [7, 13]. DNMT1 is the major DNMT that expressed ubiquitously [31] and has important role in tumorigenesis by silencing tumor suppressor genes by hyper-methylation [32, 33]. Since DNMT1 has been reported to be up-regulated in many human cancers [34], compounds inhibiting DNMT1 are currently under active development [32, 33]. High levels of acetyl-STAT3 (K685) was also found in many types of cancers including melanomas, colon cancers and TNBCs [13]. In addition, acetyl-STAT3 (K685) was suggested to crucial for promoter methylation of tumor suppressor genes such as STAT1, p53, SOCS3, SHP-1, and p16 (CDKN2A) in mouse embryonic fibroblasts, A2058 melanoma cell, and HCT116 colon cancer cell [7, 13]. In the present study, the functional consequence of disrupting the STAT3-DNMT1 interaction by SH-I-14 was re-expression of TS genes including VHL and PDLIM4 through de-methylation of their promoter DNAs. SH-I-14 induced de-methylation of these genes’ promoters near completely within a 48 hr post-treatment. The expression of VHL and PDLIM4 protein by SH-I-14 was also confirmed in both cell culture and mouse xenograft model. Re-expression of these TS genes has been reported to suppress various cancer cells’ growth both *in vitro* and *in vivo* [35–41]. Taken together, our data suggest that blocking the STAT3-DNMT1 interaction through inhibition of acetyl-STAT3 (K685) by SH-I-14 is sufficient to re-express TS genes by de-methylation of their promoter DNAs (Figure 6).

Our present study raises several questions to be addressed: 1) How does SH-I-14 facilitate the removal of acetyl group from K685 of STAT3? Although overexpression of p300 could overcome SH-I-14-mediated de-acetylation of STAT3 (K685), even 10 μM of SH-I-14 failed to inhibit acetyltransferase activity of p300 *in vitro* (data not shown). In fact, we performed a series of *in vitro* enzyme assays with purified HATs, HDACs, and SIRTs (from Reaction Biology Corp, Malvern, PA). However, SH-I-14 had no apparent activity toward these enzymes up to 10 μM concentration (data not shown). Future studies should be focused on the identification of molecular target(s) of SH-I-14. Identification of SH-I-14-binding protein(s) may provide additional target(s) to further develop potential therapeutics. 2) What is the fate of DNMT1 after dissociation from STAT3 by SH-I-14? In our present study, high concentration of SH-I-14 reduced the level of DNMT1 protein. Inhibition of STAT3-binding by SH-I-14 may affect the localization and/or stability of DNMT1. Several lines of evidences suggest that DNMT1 might be regulated by localization and/or stability. Nuclear localization signals in the N-terminal region of DNMT1 implicates that nucleocytoplasmic shuttling may be an important regulatory mechanism of DNA methylation [42]. In addition, stability of DNMT1 was increased in human cancers [43, 45] and DNMT1 is degraded by ubiquitin-dependent proteasomal degradation [45]. Interestingly, a HDAC inhibitor trichostatin A (TSA) has been reported to reduce DNMT1 protein in urothelial carcinoma cell lines [46] and reduce nuclear DNMT1 while increase cytoplasmic DNMT1 in HepG2 cell [47]. However, the effect of STAT3-interaction on the stability of DNMT1 has not been revealed yet. 3) What kinds of genes are re-repressed by SH-I-14 through de-methylation? Since DNMT1 is not a DNA-binding protein, DNA binding proteins recruit DNMT1 by protein-protein interaction to specific DNA sequences and determine the set of genes to be regulated by these protein complexes. Although recent reports demonstrated that STAT3 facilitate DNMT1 binding to several genes’ promoter [7, 13], currently there is no detailed study on the specific DNA sequences for STAT3-DNMT1 complex. As a probe to disrupt STAT3-DNMT1 interaction, SH-I-14 may provide valuable tool to determine the specific sequence for STAT3-DNMT1-DNA binding and the set of methylated genes by STAT3-DNMT1 complex. Since the method used to measure DNA methylation in this study is based upon restriction enzyme cleavage and the number of targets tested is limited, further study is needed to measure the global methylation pattern in the absence or presence of SH-I-14. 4) We cannot exclude additional negative regulation of STAT3 function by SH-I-14 on the suppression of TS genes. In the present study, we found that SH-I-14 could not disrupt STAT3^K685Q^-DNMT1 interaction while reduced the phospho-STAT3^K685Q^ (Y705) in PC-3 cells. We also found that SH-I-14 induced the expression of PTPN6/SHP-1 [20] and SOCS3 (data not shown), which negatively regulated phospho-STAT3 [3, 48] in TNBC cells. These results suggest possible alternative repression of PTPN6/SHP-1 expression by STAT3. Since DNMT1 functions to maintain DNA methylation patterns by recognizing hemimethylated CpG sequences after DNA replication [49], it might require additional time for DNA replication after inhibition of DNMT1 to sufficiently de-methylate DNA for re-expression of suppressed TS genes. As an example, it has been reported that maximal DNA de-methylation was achievable after 48 hr treatment with DNMT inhibitors, 5-azacytidine or 2’-deoxy-5-azacytidine (decitabine) [50, 51] and the mRNA expression of p16 TS gene was detected beginning 36 hr after decitabine-treatment in T24 bladder carcinoma cells [51].

In conclusion, in this research work we described a potent inhibitor of acetyl-STAT3 (K685). We confirmed that our novel inhibitor SH-I-14 could reduce the acetylation of STAT3 at K685 residue. We further found that SH-I-14 disrupted STAT3-DNMT1 interaction because of its de-acetylation of STAT3, which resulted in re-expression of tumor suppressor genes such as VHL and PDLIM4 through de-methylation of their promoter regions. Our data suggest that targeting acetylation of STAT3 (K685) by small molecule inhibitors is a plausible new approach to treat human cancers. Since both acetylation of STAT3 and the level of DNMT1 are known to be elevated in a broad range of human cancers, it is likely that the STAT3-DNMT1 complex may participate the repression of TS genes by DNA methylation in various cancers. Future profiling of acetyl-STAT3 and DNMT1 status in human cancers may provide an alternative therapeutic opportunity with a distinct mechanism of targeting the STAT3-DNMT1 interaction.

## MATERIALS AND METHODS

### Cell lines and reagents

All cell lines, except for SUM1315MO2 and SUM149PT, were obtained from the Tissue Culture Shared Resource of Georgetown University Medical Center. The reagents for cell culture were purchased from Invitrogen (Carlsbad, CA), Lonza (Basel, Switzerland) or Cellgro (Manassas, VA). HS578T, MDA-MB-231, MDA-MB-468, and HEK293T cells were maintained in DMEM containing 10% (vol/vol) heat-inactivated FBS (HI-FBS; HyClone, Logan, UT; or Omega Scientific, Inc. Tarzana, CA) and 100 units/ml penicillin/streptomycin. SUM1315MO2 and SUM149PT were maintained according to manufacturer’s recommendations (Asterand, Detroit, MI). Viable cells were monitored by the Luna Automated Cell Counter (Logos Biosystems, Gyunggi-do, Korea). AZD1480 was purchased from Selleck Chemicals (Houston, TX) and CP690550 was obtained from LC Labs (Woburn, MA). Stock solutions of compounds were made at 10 mM concentration in dimethyl sulfoxide (DMSO) and stored at -20°C in small aliquots.

### Synthesis of SH-I-14

SH-I-14 was synthesized as described previously [20].

### Plasmid DNAs and transfection

GFP-STAT3 was described elsewhere [11]. GFP-STAT3 (K685Q) and GFP-STAT3 (K685R) were constructed by mutagenesis and the mutations were confirmed by sequencing (Genewiz, South Plainfield, NJ). pRC/CMV-STAT3 was a kind gift from Dr. J. Bromberg (MemorialSloan-KetteringCancerCenter, Weill Cornell Medical College, New York, NY) and FLAG-p300 was obtained from Dr. T. Imamura (The JFCR Cancer Institute, Tokyo, Japan). Transfection was performed by Lipofectamine 2000 (Invitrogen) as recommended by manufacturer.

### Cell proliferation assay

MTT (3-(4,5)-dimethylthiazol-2-yl)-2,5-diphenyl tetrazolium bromide) assay was performed as described previously [20].

### Western blot and immunoprecipitation analysis

Western blot analysis was performed as described [20]. Antibodies used in this study were as follows: Acetyl-STAT3 (K685) (#2523), phospho-STAT3 (Y705) (#9145), STAT3 (#4904), and DNMT1 (#5032) from Cell Signaling (Danvers, MA);PARP (556494) and VHL (55647) from BD Biosciences (San Jose, CA); PDLIM4 (NBP1-80833) from Novus Biologicals (Littleton, CO); and FLAG (M2) and β-actin (A1978) from Sigma-Aldrich (St. Louis, MO). Immunoprecipitation was performed as described previously [50]. Desitometric analysis was performed by ImageJ [20].

### DNA methylation assay

Genomic DNA was isolated from HS578T and MDA-MB-231 treated with 0.3 μM of SH-I-14 for 48 hr using DNeasy Blood & Tissue kit (QIAGEN, Valencia, CA) according to manufacturer’s protocol. CpG island DNA methylation was analyzed by EpiTect Methyl II PCR array system (QIAGEN) following manufacturer’s instruction. In brief, genomic DNA was digested by methylation-dependent or/and methylation-sensitive enzymes at 37°C for overnight. Following digestion, DNA was analyzed by quantitative realtime-PCR withEpiTect Methyl II PCR array system or predesigned VHL or PDLIM4 primers (QIAGEN). Percentage of methylation and un-methylation were calculated by data analysis tool provided by QIAGEN.

### Quantitative realtime-PCR (qRT-PCR)

The qRT-PCR was performed as described [52]. The primers used in this study were synthesized from Sigma Genosys (St. Louise, MO, USA) with following sequences: VHL, forward 5´- GCG TCG TGC TGC CCG TAT G-3´ and reverse 5´- TTC TGC ACA TTT GGG TGG TCT TC -3´; PDLIM4, forward 5´-ACT TCA GCG CGC CCC TCA CCA TCT CAC-3´ and reverse 5´- TCT AGC ATG CCC TGC AAG TAG CGG AAG G-3´; ARHI, forward 5’-TCT GCC CGC CCT GCT TAT -3’ and reverse 5’- TTG CCG TCG CCA CTC TTG -3’; GAPDH, forward 5’- GTA TGA CAA CGA ATT TGG CTA CAG -3’ and reverse 5’-AGC ACA GGG TAC TTT ATT GAT GGT -3’.

### Cell cycle analysis

Cells were treated with either DMSO or SH-I-14 for 24 hr. The attached cells were harvested by trypsinization and combined with floating cells in the culture media. After washing by phosphate-buffered saline (PBS), the cells were fixed with 70% ethanol at -20˚C. Flow cytometric analysis of propidium iodide (PI)-stained cells was performed with a FACS Calibur flow cytometer (Becton-Dickinson, CA) at the Flow Cytometry and Cell Sorting Shared Resource of Georgetown University Medical Center.

### *In Vivo* xenograft tumor models

Animal use procedures were approved by the Institutional Animal Care and Use Committees of Georgetown University Medical Center. MDA-MB-231 cells (2.5 × 10^6^ cells/mouse) mixed with Matrigel (BD Biosciences, San Jose, CA) were injected subcutaneously into the flank of male athymic nude (*Foxn1*^nu^) mice of 6-week age (Harlan Laboratories, Frederick, MD). SH-I-14 was dissolved in DMSO at concentration of 30 mg/ml. For injection of compound, DMSO or SH-I-14 was diluted in a 1:1 mixture of PBS and injection solution. Injection solution was made as described previously [20]. Diluted SH-I-14 (10 mg/kg) or DMSO was administered into peritoneal cavity of mouse with 3 times per week. The body weights and tumor sizes were measured before every injection. Tumor sizes were measured using a digital caliper and tumor volumes were calculated using the formula, [volume (mm^3^) = width (mm) × length (mm) × height (mm) / 2].

For western blot analysis of protein markers, xenograft mice were established as described above. SH-I-14 (10 mg/kg) or DMSO was administered into peritoneal cavity of mouse daily for 5 days. Mice were euthanized at 1 hr after last administration of drug. Tumors from each mouse were lysed and subjected to western blot analysis.

### Statistical analysis

To compare two groups of interest, the Turkey’s multiple comparison test (ANOVA) was applied for statistical analysis. * indicates *P* < 0.05; ** indicates *P* < 0.01; and *** indicates *P* < 0.001.

## SUPPLEMENTARY MATERIALS FIGURES


