# Supplementary material for: Disruption of STAT3-DNMT1 interaction by SH-I-14 induces re-expression of tumor suppressor genes and inhibits growth of triple-negative breast tumor
Source: Oncotarget. 2015 May 9;8(48):83457–68. doi: 10.18632/oncotarget.4054 (PMC5663528; doi:10.18632/oncotarget.4054)
Supplement: Supplementary file 1 [file oncotarget-08-83457-s001.pdf]

Disruption of STAT3-DNMT1 interaction by SH-I-14 induces re-expression of tumor suppressor genes and inhibits growth of triple-negative breast tumor

SUPPLEMENTARY MATERIALS

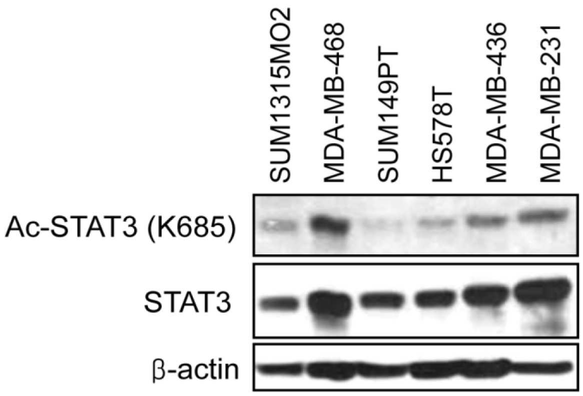

Supplementary Information, Figure S1 The level of Ac-STAT3 and STAT3 was determined by western blot analysis. The cell lysates from the exponentially growing cells were subjected to western blot analysis. β-actin was used as a loading control.

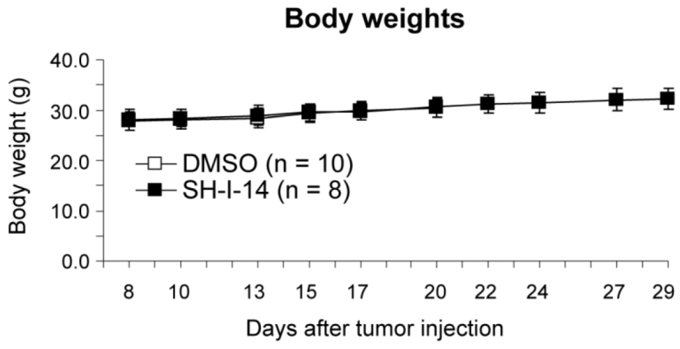

Supplementary Information, Figure S2 SH-I-14 did not affect the body weights of xenograft mice. Body-weight of mice in Figure 5A are presented as mean ± SD.
